# Snake W Sex Chromosome: The Shadow of Ancestral Amniote Super-Sex Chromosome

**DOI:** 10.3390/cells9112386

**Published:** 2020-10-31

**Authors:** Worapong Singchat, Syed Farhan Ahmad, Nararat Laopichienpong, Aorarat Suntronpong, Thitipong Panthum, Darren K. Griffin, Kornsorn Srikulnath

**Affiliations:** 1 Laboratory of Animal Cytogenetics and Comparative Genomics (ACCG), Department of Genetics, Faculty of Science, Kasetsart University, 50 Ngamwongwan, Chatuchak, Bangkok 10900, Thailand; worapong.si@ku.th (W.S.); farhan.phd.unesp@gmail.com (S.F.A.); nararat.l@ku.th (N.L.); aorarat.sun@ku.th (A.S.); thitipong.pa@ku.th (T.P.); 2 Special Research Unit for Wildlife Genomics (SRUWG), Department of Forest Biology, Faculty of Forestry, Kasetsart University, 50 Ngamwongwan, Chatuchak, Bangkok 10900, Thailand; 3 School of Biosciences, University of Kent, Canterbury CT2 7NY, UK; D.K.Griffin@kent.ac.uk; 4 Center for Advanced Studies in Tropical Natural Resources, National Research University-Kasetsart University, Kasetsart University, (CASTNAR, NRU-KU, Thailand), Bangkok 10900, Thailand; 5 Center of Excellence on Agricultural Biotechnology (AG-BIO/PERDO-CHE), Bangkok 10900, Thailand; 6 Omics Center for Agriculture, Bioresources, Food and Health, Kasetsart University (OmiKU), Bangkok 10900, Thailand; 7 Amphibian Research Center, Hiroshima University, 1-3-1, Kagamiyama, Higashihiroshima 739-8526, Japan

**Keywords:** chromosomal rearrangements, evolution, genome, next-generation sequencing, sex determination, repeat elements

## Abstract

Heteromorphic sex chromosomes, particularly the ZZ/ZW sex chromosome system of birds and some reptiles, undergo evolutionary dynamics distinct from those of autosomes. The W sex chromosome is a unique karyological member of this heteromorphic pair, which has been extensively studied in snakes to explore the origin, evolution, and genetic diversity of amniote sex chromosomes. The snake W sex chromosome offers a fascinating model system to elucidate ancestral trajectories that have resulted in genetic divergence of amniote sex chromosomes. Although the principal mechanism driving evolution of the amniote sex chromosome remains obscure, an emerging hypothesis, supported by studies of W sex chromosomes of squamate reptiles and snakes, suggests that sex chromosomes share varied genomic blocks across several amniote lineages. This implies the possible split of an ancestral super-sex chromosome via chromosomal rearrangements. We review the major findings pertaining to sex chromosomal profiles in amniotes and discuss the evolution of an ancestral super-sex chromosome by collating recent evidence sourced mainly from the snake W sex chromosome analysis. We highlight the role of repeat-mediated sex chromosome conformation and present a genomic landscape of snake Z and W chromosomes, which reveals the relative abundance of major repeats, and identifies the expansion of certain transposable elements. The latest revolution in chromosomics, i.e., complete telomere-to-telomere assembly, offers mechanistic insights into the evolutionary origin of sex chromosomes.

## 1. Introduction

A fundamental aspect of the life history of sexually reproducing organisms is the fusion of haploid gametes produced by meiosis through the mechanisms of independent assortment and genetic recombination of chromosomes from two parental genomes to form a zygote. This contributes to the phenotypic diversity at population and species levels arising from natural selection during the course of evolution. Sex determination is the process by which organisms develop as either male or female, and it exhibits remarkable mechanistic diversity and turnover among species. The mechanisms range from environmental sex determination (ESD), where sexes do not differ in genotype, to genotypic sex determination (GSD) resulting from homomorphic to highly differentiated heteromorphic sex chromosomes as either male heterogametic (XX/XY) or female heterogametic (ZZ/ZW) [1,2,3].

Amniotes diverged into two major lineages comprising Synapsida, including all living mammals, and Sauropsida, including all extant non-avian reptilian and avian species, with substantial variation in sex determination mode [4,5]. Sex chromosomes classically evolve from a pair of autosomes (proto sex chromosomes) after one autosome acquires a sex-determining locus [6]. This locus is located on the Y or W sex chromosome and is restricted to a single sex, which affects subsequent processes in the adjacent region as sexually antagonistic genes. To produce a novel allele or gene, a genetic variant must gain control over the sex determination cascade, which is subject to a master sex-determining gene [6,7]. This might have occurred through a point mutation causing gene knockout and loss-of-function, or creating a novel function and regulatory change [8,9,10,11,12,13]. The master sex-determining gene can act in a dominant fashion on the Y or W sex chromosomes, where one copy is needed to determine maleness (on a Y sex chromosome) or femaleness (on a W sex chromosome), such as *SRY* in mammals or in a dose-dependent manner on the X or Z sex chromosomes, where two functional copies are needed for femaleness (on the X sex chromosome) or maleness (on the Z sex chromosome) [6,7]. Regions around this master sex-determining locus progressively stop recombination with their respective homologous regions on the X/Z counterparts [6,7]. This suppression of recombination might be involved with the selective advantage and preservation of linkage disequilibrium between sex-determining and sexually antagonistic genes, leading to multiple formations of evolutionary strata in sex chromosome evolution and differentiation [6,14,15,16,17,18,19]. 

Cessation of recombination triggers structural changes, predominantly on the Y or W sex chromosomes, including accumulation of deleterious mutations, degradation of gene content, accumulation of repeats, heterochromatinization, and changes in gene expression [20,21,22,23,24,25,26,27,28,29,30,31,32]. Deleterious mutations might accumulate in the nonrecombining region through Muller’s ratchet or genetic drift, causing Y or W genes to lose their function or disappear altogether [33]. Simultaneously, strong selection acting on the sex-determining region can induce background selection, genetic hitchhiking, and selective sweeps that reduce genetic variability in the adjacent regions [34]. Chromosomal inversions surrounding the master sex-determining region probably occurred on the Y or W chromosomes, thereby preventing chromosome pairing and crossing over with the homologous X- or Z-linked inverted regions, as observed in chicken and Japanese quail [35,36,37,38]. The degree of divergence between X and Y or Z and W sex chromosomes is independently observed across amniote lineages with remarkable variation [39]. Synonymous substitution rates of XY (or ZW) gametologous genes, which are homologous genes located in the nonrecombining region of differentiated sex chromosomes, can be used to trace the evolutionary history of sex chromosomes [14,16,32,40,41,42,43,44,45,46,47,48]. 

Despite considerable research efforts and recent advances in omic technologies, prediction of the ancestral and transition states between particular sex determination modes and sex chromosomes in amniotes remains uncertain [18,49,50,51]. The ancestral state might be ESD or polygenic sex determination, changing to GSD later on [18,52,53,54,55]. By contrast, it might include the presence of multiple transitions from GSD to ESD. Sex chromosomes evolved independently multiple times within amniotes and remained notably stable after their emergence in mammals, birds, and many lineages of reptiles [16,17,18,56,57]. This scenario is supported by evidence of the same linkage homology blocks that perform the role of sex chromosomes in several amniote lineages, or by unrelated sex chromosomes sharing partial linkage homologies across distantly related groups [18,25,27,28,30,31,32,58]. This was probably caused by multiple random selections from a limited number of linkage homologies, or a stronger tendency for a linkage homology to be co-opted owing to its gene content, particularly as a result of enrichment of the genes involved in gonad differentiation, and the possibility of homologous sex-determining systems [7]. The molecular machinery of the sex determination pathway is observed across ESD amniote lineages, which concurs with independent co-option of the same epigenetic process [59]. Interestingly, recent comparative genomic analyses indicate that the majority of the squamate reptile chromosome 2 (SR2) and the snake W sex chromosomes share partial sex chromosomal linkage homologies with sex-related elements of other amniotes, despite their apparent diversity of sex-determining mechanisms [18,25,27,28,30,31,32,58]. Hypothetically, the SR2 and snake W sex chromosomes may have been part of a larger ancestral amniote super-sex chromosome with a GSD system that subsequently split into many sex chromosomes across several amniote lineages by multiple chromosomal rearrangements such as fission [27,28,30]. This hypothesis suggests an incredible diversity of sex-determining systems, raising questions for many models, including: (i) whether several lineages co-opted the same chromosome pair, or at least parts of them, to function as sex chromosomes?; (ii) whether these co-options resulted from the lack of alternatives, with only a limited number of chromosomes in the ancestral karyotype to form sex chromosomes, or certain unique characteristics of these chromosomes found in SR2 and snake W sex chromosomes, and particularly if the content of genes involved in gonad differentiation predisposed certain chromosomes to become sex chromosomes?; and (iii) what drives some sex chromosomes to be maintained over millions of years and differentiate fully, while others are replaced by new sex-determining chromosomes before differentiation has occurred?

Here, we review evidence pertaining to different sex chromosomal profiles in amniotes obtained from chromosomics and show that correlation with snake W sex chromosomes is a relict of an ancestral super-sex chromosome. Using data sourced from a recent near-complete chromosome-level assembly of the Indian cobra (*Naja naja*) genome [60], we also report the comparative repeatomic landscape of Z and W chromosomes and highlight the genomic abundance of major repeated elements on sex chromosomes. Evolutionary dynamics of repeat-mediated sex chromosome formation are also discussed.

## 2. Turnover of Sex Chromosomes in Amniotes

**Figure 1:**
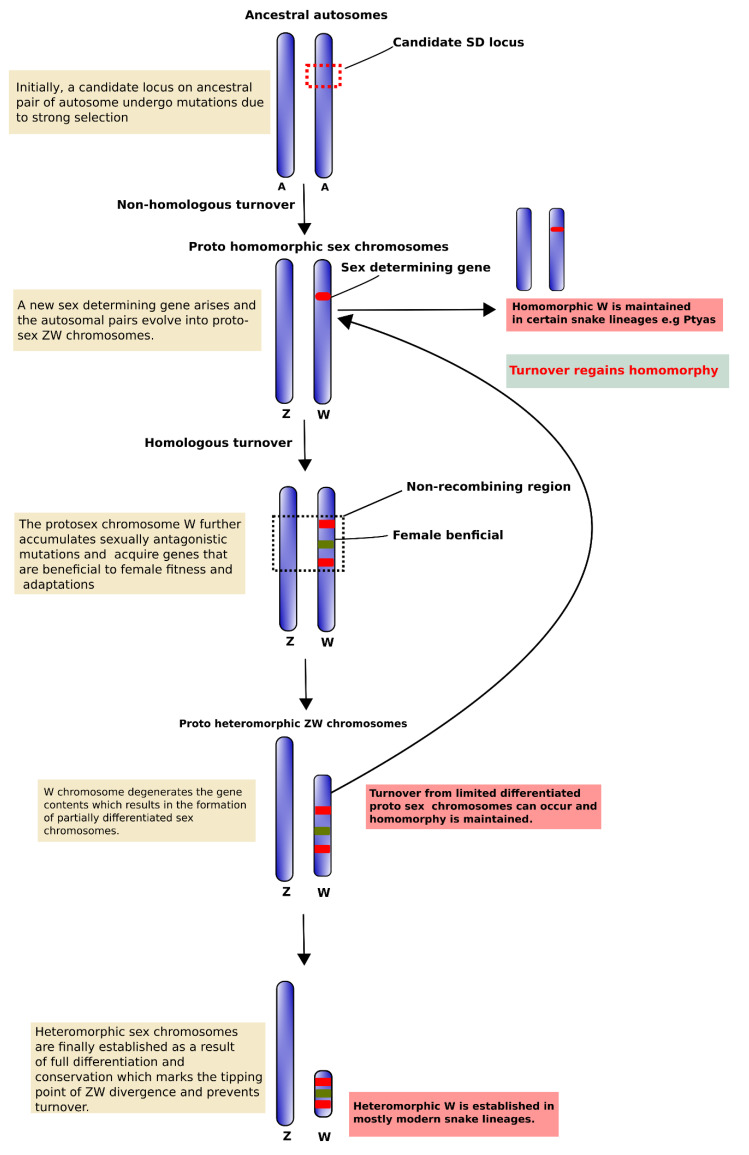
Schematic diagram of different phases in ZW sex chromosome evolution. We propose a hypothetical evolutionary model to illustrate the origin and evolution of ZW sex chromosomes. First, owing to strong selection of an evolutionary hotspot region, an ancestral autosomal pair undergoes mutation to become a sex determination region, and transformation into homomorphic proto-sex chromosomes. This is followed by heteromorphic differentiation resulting in formation of a proto-W chromosome with cessation of recombination and gain of female beneficial sequences for fitness and adaptation. The proto-W chromosome subsequently undergoes structural changes, such as rearrangements, gene degradation, repeat accumulations, and heterochromatinization, to form a neo-ZW chromosome system with limited differentiation. In some cases, during this stage turnover cycles might convert the partially differentiated heteromorphic sex chromosomes into homomorphic sex chromosomes, as in certain snake species, such as Ptyas species. To achieve full heteromorphy the neo-ZW chromosomes escape this evolutionary trap, and the young W chromosome undergoes severe degeneration with lineage-specific sequence variation and evolves into a mature and stable sex chromosome.

Sex chromosomes carry important sex-determining genes and/or genes that specifically influence male or female fitness, and may have facilitated their recruitment for sex determination [6,7]. Sex chromosome turnover occurs when the existing master sex-determining gene physically moves onto an autosome and retains its control over sex determination [61]. Sex determination systems and/or sex chromosomes have evolved independently numerous times, with frequent turnover from one system to another, exhibiting both inter- and intra-specific variation across many species of amniotes, whereas the X and Y (or Z and W) chromosomes of mammals and birds are conserved [2,40,51,56,58,62,63,64,65,66,67,68]. There are two possible explanations for the emergence of new sex-determining genes and sex chromosomes across amniote species [2,69]. First, when a new sex-determining locus arises on an autosome, it converts the autosome into a ‘proto-sex-chromosome’, and the ancestral sex chromosome reverts to an autosome (Figure 1) [70,71]. Turnover can occur when a new master sex-determining gene arises de novo on an autosome (termed ‘non-homologous turnover’) [61,72,73]. The emergence of a new master sex-determining locus can have very different consequences depending on how it interacts with the previous sex determination system [61,73]. If the new sex-determining locus was associated with a gain in fitness, turnover is more likely to result in different sex chromosomal linkages between species. By contrast, when a new sex-determining gene arises on the existing sex chromosome (termed ‘homologous turnover’) [61,73], turnover between XY and ZW determination systems on the same chromosome arises in the course of evolution. Caenophidian snakes share the same ancestral ZW chromosomes, with varying degrees of W degeneration; however, pythons have an XY system, leading to the emergence of a new sex-determining locus, although only a few specimens have been examined [41,47]. The question of how and why these turnovers arise remains unclear but is assumed to result from sexual conflict, genetic drift, and mutation accumulation [73,74,75,76,77,78,79,80]. Second, autosomes can translocate (by simple translocation, centric fusion, or insertion) to sex chromosomes and create ‘neo-sex-chromosomes’ as observed in stickleback and black muntjac [6,81,82]. In the fusion process between sex chromosomes and autosomes, chromosomes harboring such genes may often be involved in the formation and turnover of sex chromosomes, forming neo-sex-chromosomes [6,83]. It is likely that sex-specific selection pressures (including sexual antagonism) are the primary evolutionary contributors to sex determination pathways, evolutionary turnover in sex chromosomes, and the fixation of neo-sex-chromosomes [18,75,76,84,85,86,87,88,89].

Key questions include ‘why do some lineages maintain and conserve sex chromosome/sex determination?’ and ‘why do other lineages show frequent recurrent turnover?’ The answers might be informed by the mechanism of the ‘evolutionary trap’ hypothesis [54]. Sex chromosomes may undergo cycles of turnover by default unless a tipping point of differentiation is crossed. Sex chromosomes then are stably maintained and fully differentiate, which prevents frequent transition from GSD to TSD or turnover to different GSD systems [40,54]. The transition between GSD and TSD requires traversing a group of fitness-related genes, where individuals are produced carrying suboptimal or lethal WW or YY genotypes. Sex chromosome turnover involves the fixation of a new sex-determining locus in the population, varying the effective population size of the species [73,79,90,91]. By contrast, reptiles possess homomorphic sex chromosomes that appear to be evolutionarily young, owing to frequent turnover [3,92]. The transition from GSD to ESD, as well as turnover of sex chromosomes within GSD, requires an intermediate step of sex reversal, producing individuals with a mismatch between phenotypic and ancestral genotypic sex [51]. Sex-reversed individuals should lack a specialized sex-specific combination of sex chromosomes in lineages with differentiated sex chromosomes and thus show lower fitness. Such sex-reversed amniotes with differentiated sex chromosomes are infertile or possess atypical sex-specific phenotypes [93,94,95]. Homomorphic sex chromosomes are maintained by occasional XY or ZW recombination in sex-reversed XY females or ZW males, known as the ‘fountain of youth’ hypothesis. This is possible if recombination suppression is independent of phenotypic sex assignment [96,97,98]. This might enable escape from the trap and independent evolution in the lineage. Pleurodonts and the sister group corytophanids, a family of iguanian lizards, harbor different partial sex chromosomal linkage groups within each lineage [99,100,101]. However, the tendency for recurrence of sex chromosomal groups might result in homoplasy. New data emerging from non-model sex chromosome systems may provide interesting exceptions to the hypothesis on how sex chromosomes originate and evolve, and suggest diversity in the process not previously acknowledged. Systematic differences between amniote lineages and their frequency of acquisition of stable sex chromosome/sex determination require further investigation to obtain more conclusive evidence.

## 3. Sex Chromosomal Linkage Homology in Relation to SR2 and Snake W Sex Chromosome

Comparison of genome assemblies and chromosome maps among amniotes have revealed a high degree of linkage homology and elucidated the process of chromosomal rearrangement over millions of years [20,21,25,27,28,30,102,103,104,105,106,107,108,109,110,111,112,113,114]. In a few cases, sex chromosomes share homology among some amniote lineages; however, genome sequence analyses and cross-species chromosome mapping have revealed that unrelated sex chromosomes share linkage homologies across distantly related taxa, and might involve genomic regions orthologous to SR2 and the snake W sex chromosome [18,25,27,28,30,58,60]. These overlaps of partial sex chromosomal linkage homology may be part of a hypothetical ancestral super-sex chromosome (Box 1). Portions of an ancestral super-sex chromosome probably exist in amniotes, with multiple chromosomal rearrangements, such as fissions or insertions, as evolutionary sources of various sex chromosomal linkages [18,25,27,28,30,31,32,58,115]. Under this concept of a super-sex chromosome, sex-specific nonrecombining regions of Y or W sex chromosomes are likely to be ‘super-segments’, enabling adaptation to sex-specific functions or sex-biased expression [33,116]. Co-localization of these genes/regions might reflect the co-option of particularly favorable genes/segments. The same sex-determining genes, as orthologous or paralogous states, have been used repeatedly in distantly related amniotes, such as *DMRT1*, *SOX3*, or *AMH* in a sex-determining function [16,117]. Particular linkage homology is often associated with sexual development in distantly related amniote lineages because of genetic hitchhiking [118].

Alternatively, the occurrence of a super-sex chromosome might reflect group sex-determining gene interactions. The majority of sex-determining genes in one species are part of the conserved sex-determining network in all amniote lineages. Several genes have been independently recruited to the first step of the sex-determining pathway in different species, where each is probably necessary and sufficient for sex determination [2,119]. Their physical proximity may facilitate biochemical interaction of the products of these genes to bring about sex determination. In some cases, such as *Drosophila*, sex is determined according to the ratio of X chromosomes to autosome sets. Key genes involved in the sex-determining pathway include *Sxl*, *tra*, and *dsx*, and expression of these genes is regulated by several transcription factors encoded on the X chromosome [120,121,122]. Another plausible hypothesis concerns chromosome territory, which could make their physical translocation more likely, as in the case of translocations between chromosomes bearing nucleolus organizing regions (NORs) [123]. Chromosomes occupy highly conserved territories in somatic cells of mammals, birds [124,125,126], and mammalian germ cells [127]. The positions of these territories are associated with the gene content of chromosomes; sex chromosomes with a low gene density are more frequently located at the periphery [128,129]. In addition to several models for the origin of a super-sex chromosome, an underlying principle of sex determination in amniote lineages is the sharing of linkage homology. Sequences such as repeats were once linked in a super-sex chromosome that was broken up by different means. Many changes to genomic content occur once an autosomal pair becomes a sex chromosome pair. As well as deletion, selection of sex-specific traits on the sex-specific chromosome (Y or W) and changes in the genomic content of the partner sex chromosome (X or Z) reflect the hemizygous state in one sex [6,130,131]. Particular sequences, such as 18S–28S ribosomal RNA genes, may play roles in sex chromosome regulation or create novel sex chromosomes [18,58,132]. Opossum and kangaroo sex chromosome pairs have independently fused with a segment carrying the NOR, whereas platypus sex chromosomes are frequently arrayed around the nucleolus during meiosis, which brings them into close proximity to the NOR-bearing chromosome 6 that shows homology with the human X-conserved region [133,134]. Proximity to the site of RNA synthesis might facilitate epigenetic processes involving long noncoding RNAs [135]. Minimally differentiated XY chromosomes are observed in three cryptodiran turtles (*Staurotypus crassicollis*, *S. triporcatus*, and *S. salvinii*), in which the Y chromosomes are smaller than the X chromosomes owing to a difference in copy number of 18S–28S rRNA genes [136,137]. SR2 is highly conserved among squamate reptiles [58,102,103,132,138], and NORs are generally located on a pair of microchromosomes or chromosome 2 in iguanas and some snakes [139,140]. The NORs are located on the ZW microchromosomes in the bearded dragon (*Pogona vitticeps*), which share a common ancestry with SR2 [3,58,109,115,132,141].

From a different perspective, recent studies of many amniotes have revealed a striking difference of the gene and repeat content of their Y/W sex chromosomes, with substantial disparity even between closely related species [16,17,47,51,142,143,144,145,146,147,148,149,150]. This is despite the prolonged stability of sex determination systems in these lineages and the extensive between-species homology of their X/Z-specific gene contents [17,42,151]. However, genomic regions of snake W sex chromosomes show substantial homology with sex chromosomal linkage homologies and repeat content in amniotes [25,27,28]. Singchat et al. [25,27,28] asserted that 16 bacterial artificial chromosomes (BACs) showing partial homology with sex chromosomes of several amniotes were mapped on the heterochromatic W sex chromosomes of different species, including Siamese cobra (*Naja kaouthia*), Russell’s viper (*Daboia russelii*), and the common tiger snake (*Telescopus semiannulatus*), based on hybridization signals such as repeats. In comparison, two chicken BACs located on *Gallus gallus* chromosome Z (GGAZ) that showed a high abundance of the long interspersed nuclear element (LINE) and long terminal repeat (LTR) transposable elements (TEs) were mapped on SR2 and the snake W sex chromosome [25,27,28]. This suggests that repeats on the snake W sex chromosome also share sex chromosomal linkage homology to SR2 and GGAZ, and the snake W sex chromosome might include a genomic region involving sex chromosome conformation in amniotes. The Y or W sex chromosomes showing accumulation of satellites and amplification of telomeric or microsatellite repeats [(GATA)_n_, (AAGG)_n_, (AATC)_n_, and (ACAG)_n_] are commonly observed in snake W chromosomes and in other amniotes [20,22,23,24,25,29,115,152,153,154,155]. One microsatellite amplified on the W sex chromosome in several caenophidian snakes is the banded krait minor satellite (Bkm), which consists of a microsatellite repeat motif (AGAT)_n_ or (GACA)_n_ sequence, and is associated with the degree of ZW differentiation [156]. This might result from rapid and independent amplification of repeats on W sex chromosomes, and suggests that frequent amplification of the repeats has a structural role in heterochromatinization and promotes further sex chromosome differentiation [25,27,28,29,30]. Amplification of repeats has occurred independently in each lineage and might represent convergent sex chromosomal differentiation among amniotes [18,25,30]. Interestingly, bird and snake W sex chromosomes share blocks of three repeats (Bkm repeats, 18S–28S rRNA-related repeats, and DMRT-related repeats) [23]. This suggests that repeats are shared partially between the sex chromosomes of chicken and snakes, and supports the hypothesis that SR2 and the snake W sex chromosome were associated with a larger ancestral amniote super-sex chromosome [18,25,27,28,30,58]. Many studies have identified convergent genomic patterns in independently formed sex chromosomes [34,157], and causes of the repeated origins of these unique regions of the genome have been suggested [6]. Amplified repeats were possibly retained in the sex chromosomes of an amniote common ancestor, and subsequent reshuffling led to the appearance of sex chromosomes in each lineage. Convergent evolution of sex chromosomes across distantly related taxa might lead to genomic elements, such as repeats, which are particularly adept in a sex-determining role. However, the majority of repeats or genomic regions are more likely to be associated with snake W sex chromosomes [25,27,28]. Most of these explored orthologous regions have been cytogenetically mapped to better understand candidate sequences such as BACs or other repeats; however, further chromosomic level studies will elucidate the possible occurrence of linked genes in shared chromosomal regions [30,31,32,49].

## 4. Repeats: A Driver for Sex Chromosome Conformation after the Split of an Ancestral Amniote Super-Sex Chromosome

During the process of sex chromosome differentiation, heterochromatin is enriched at repeats (TEs and satellites), and its loss can result in de-repression and mobilization of silenced TEs. The number of repeats can differ substantially between sexes owing to the presence of a highly repeated (and normally poorly assembled) Y or W sex chromosome in the heterogametic sex individual [29,92,155,158]. Transposable elements are located at the boundaries of recombining and nonrecombining regions, which suggests their causal role [159,160]. Insertion of TEs near the sex-determining locus can act to suppress recombination by creating a divergence between sex chromosomes, and TEs are often assumed to accumulate following suppression of recombination. This would invoke host mechanisms to silence TEs, resulting in suppressed recombination at hotspots adjacent to TE insertions [161]. Weaker selection against the insertion of additional TEs leads to their accumulation under a lack of recombination. Moreover, TEs can promote ectopic recombination, facilitating genomic rearrangement to further suppress recombination [162]. The heterochromatic regions in amniotes are also predominantly accumulated by satellite DNA, in a class of repeats characterized by a tandem arrangement with highly repeated monomeric units longer than 100 bp, or simple repeats, such as mini- (>10 and <100 bp) and microsatellites (usually <10 bp) [163,164]. These satellites are often abundant on sex chromosomes in amniotes [25,27,28,29,30,115,165,166,167,168]. Lacertid lizards have highly differentiated ZZ/ZW sex chromosomes, and the W sex chromosome is indicated to be enriched in satellite motifs in *Acanthodactylus lineomaculatus*, *Eremias velox*, and several species from the genera *Lacerta* and *Timon* [169,170,171,172,173,174]. The primary function of the satellites is unknown; however, they may contribute to the suppression of recombination, heterochromatinization, and changes in gene expression. Different types of these repeats are randomly accumulated on sex chromosomes and largely reflect historical contingency [175,176,177,178]. The important functional role of such sequences implies that the pattern of the distribution of their accumulation should be relatively well conserved across species of the same lineage. In snake, PBI-DdeI (196 bp) satellite DNA is located in the centromeric region of the Burmese python [168]. Interestingly, PBI-DdeI satellites are frequently localized to the W sex chromosome of Siamese cobra. Localization of high copy numbers in female rather than male individuals suggests that PBI-DdeI might act as an evolutionary driver with several repeats, facilitating W sex chromosome differentiation and heterochromatinization [29]. Transposable elements and satellites may play a critical role in the early stages of recombination suppression, with the ability to shuffle genes and alter expression patterns. Repeats may simultaneously promote the turnover of sex chromosomes and sex-determining genes, initiating suppression of recombination, chromosomal rearrangements, and eventual recruitment of sex chromosomes [38].

**Figure 2:**
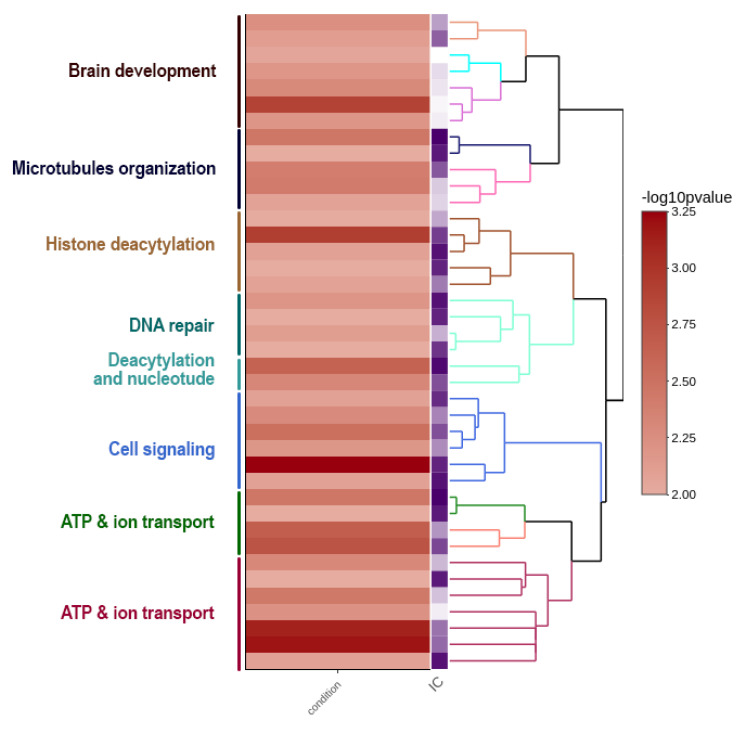
Gene ontology (GO) enrichment of annotated genes on the W chromosome of Indian cobra. Clustering heatmap plot with log_10_ (*p*-value) from functional enrichment tests and information content (IC). A higher log_10_ (*p*-value) represents a more enriched function. The results show that W chromosomes carry an enriched set of genes associated with development, histone deacetylation, signaling, and transport.

**Figure 3:**
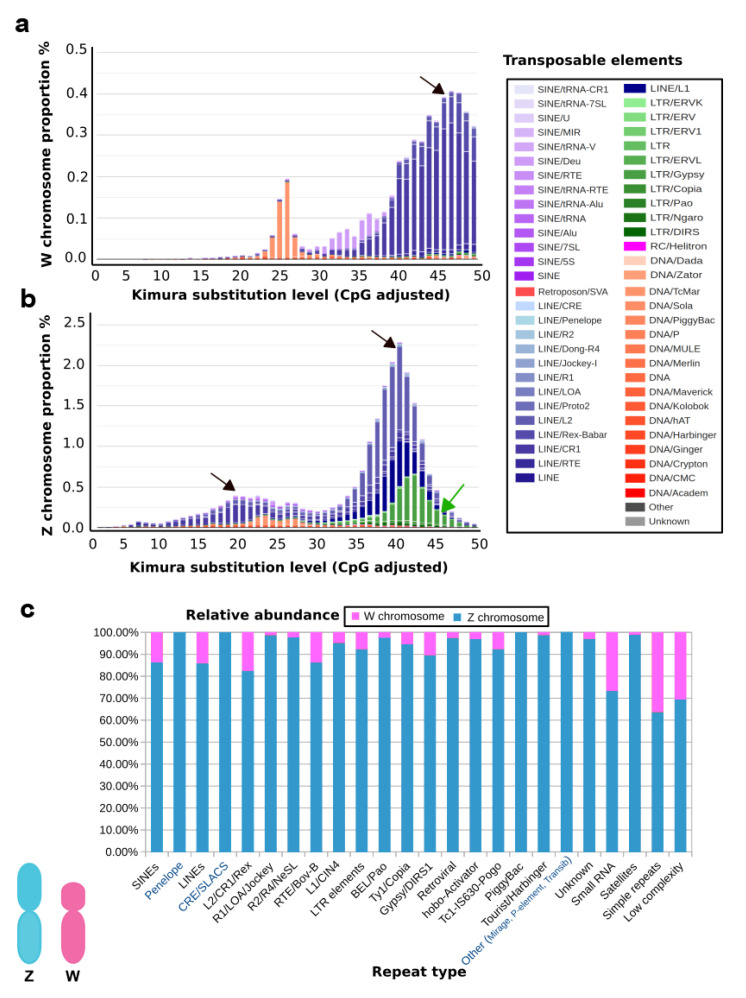
Comparative genomic characterization of repeated DNA contents between the W and Z sex chromosomes. Repeat landscape of (**a**) W and (**b**) Z sex chromosomes. Histogram plots show the degree of sequence divergence of each transposable element (TE) derived from its consensus (*X*-axis) in relation to the percentage of its copies in the total genetic contents of the chromosome (*Y*-axis). Peaks represent waves of insertion (black arrows) of elements into the sex chromosome. Older insertions of TEs are shown as a peak wave on the right side (*K*-value > 25) of the plot, whereas younger elements are depicted on the left side (*K*-value < 25). Different colors show distinct element types, as described on the right. A higher abundance of LTRs (green) of the Z sex chromosome landscape as indicated by a green arrow, is evident. The *Y*-axis percentage difference and a recent wave of expansion on the Z chromosome are evident. (**c**) Comparative analysis of Z and W localized repeat contents. Each column represents the copy number percentage stacked for the repeated element. Different proportions for Z and W sex chromosomes are indicated in blue and red, respectively. Elements with higher proportions (in blue) show expansion; those TEs present exclusively on Z chromosomes, and absent on W chromosomes are highlighted in blue text on the *X*-axis.

A well-known example is the genome of the Indian cobra, which is closely related to the Siamese cobra [179]. This genome encompasses a total size of 1.79 Gb with W and Z chromosomes spanning 52.1 Mb and 154.6 Mb, respectively [60]. This shows that the Z sex chromosome is almost three times larger than the W sex chromosome in genomic content. Ideally, the genes occurring on the W sex chromosome might play a different role in determining female-associated phenotypes, while changing female-biased selective forces might strongly affect the evolution of the W sex chromosome [180]. Although several predictions have been proposed, the W sex chromosome’s functional role is unknown, except for a few W-linked genes that have been studied mostly in birds [180]. Here, we present a functional view of the W sex chromosome of Indian cobra to corroborate the hypothesis that the W sex chromosome might be involved in multiple functions of cellular processes in addition to sex determination. The W sex chromosome harbors a diverse set of genes and, based on a gene ontology enrichment analysis (Supplementary Note 1), the W sex chromosome is enriched with genes coding for brain development, microtubule organization, histone deacetylation, DNA repair, signaling, and transport (Figure 2 and Supplementary Dataset 1). In addition, the repeat contents of Indian cobra sex chromosomes are presented (see Supplementary Note 1). The comparative repeatomic landscape of the highly repeated Indian cobra Z and W assembled chromosomes showed remarkable differences between TE abundance and the overall higher enrichment of repeats; with total repeats of 22.57% on Z chromosomes compared with 15.39% on W chromosomes. A similar pattern might be observed in other snakes with a smaller W sex chromosome [181]. The majority of TEs such as retroelements (LINEs and short interspersed nuclear elements) and DNA transposons are highly abundant on the Z sex chromosome. The most abundant elements are L2/CR1/*Rex*, which constitute 9.99% and 7.44% of Z and W sex chromosomes, respectively, with certain elements (CRE/SLACS, PiggyBac, Mirage, and *P*-elements) completely absent on the W sex chromosome (Figure 3 and Table 1). Did the Z sex chromosome become larger as the result of insertions of specific repeat elements, or did the W sex chromosome experience depletion of these elements? Chromosome mapping has indicated a high accumulation of repeats as telomeric repeats, microsatellites, satellites, and TEs on the snake W sex chromosome, with none or fewer on the Z sex chromosome [25,27,28,29]. Chicken BAC sequences mapped on the snake W sex chromosome show nonhomology to the Indian cobra W sex chromosome-level genome assembly [25,27,28,60]. Comparative repeatomic analysis supports our hypothesis that the Z sex chromosome of Indian cobra might have experienced a recent explosion of TEs that could have contributed to further gain in genetic contents. The Kimura substitution landscape TE model also indicated that the W sex chromosome contained many ancient/degenerated copies of LINEs, whereas the Z sex chromosome accumulated many recent or less divergent copies of these elements with two peaks/rounds of TE insertions (Figure 3). Collectively, these results suggest that repeats on the snake W sex chromosome might be inherited from an ancestral amniote sex chromosome with high differentiation via nonhomologous recombination, which has resulted in the evolution of heteromorphic Z and W sex chromosomes in advanced snakes [30,32]. This finding agrees with the results of BAC fluorescent in situ hybridization (FISH) mapping on the Siamese cobra, Russell’s viper, and common tiger snake [25,27,28]. These TEs may have undergone molecular degeneration, rending their identities senescent in the Indian cobra genome. A time estimation model and molecular evolutionary analyses of TE insertions could further advance our understanding and solve the complex issue of whether a recent new explosion of TEs occurred in the Indian cobra genome. Comparison of Siamese cobra and Indian cobra genomes would provide further insights into the possible occurrence of repeated elements in closely related species, and allow in-depth comparisons of repeat element density and distribution of autosomes versus sex chromosomes.

## 5. Diversity and Stability of Snake Sex Chromosomes

**Figure 4:**
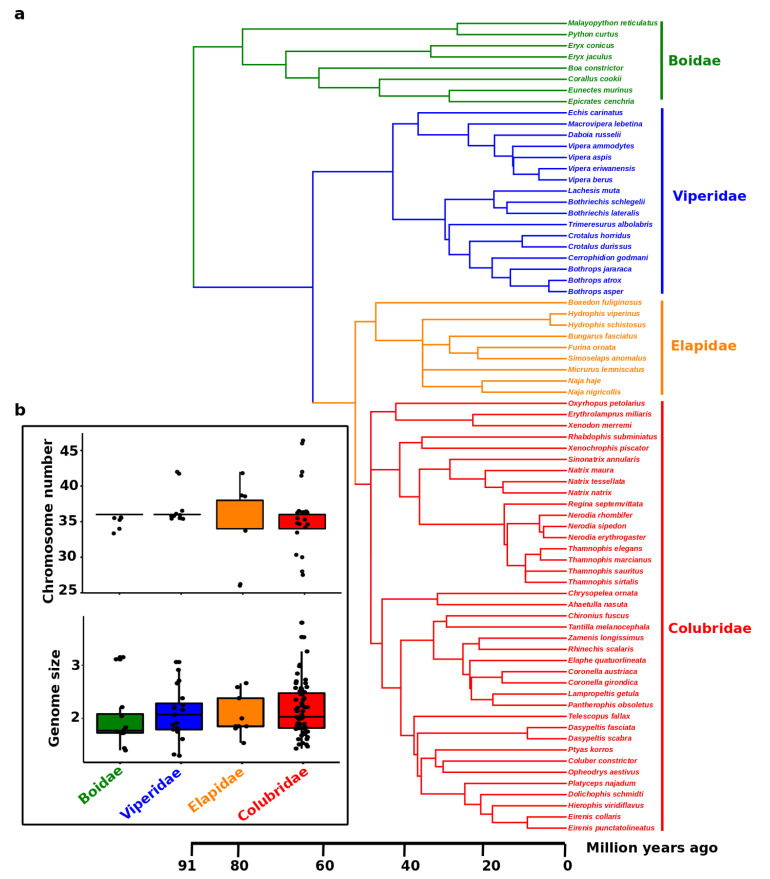
(**a**) Phylogeny of 108 snake species, with available data for karyotypes and genome size, belonging to the families Boidae, Viperidae, Elapidae, and Colubridae. (**b**) Boxplots show the distribution of chromosome number and genome size (C-value) for the four families. Each dot represents the species as given in the phylogeny. The phylogenetic tree was sourced from TimeTree databases (http://www.timetree.org) [202] and shows each species with information on chromosome number (2*n*) and genome size variation. Data were sourced from the Animal Genome Size Database (http://www.genomesize.com) [203].

Snakes represent about one-third of all reptilian species, with almost 3800 extant species classified into three major lineages: the Caenophidia, the likely paraphyletic Scolecophidia, and Henophidia [182,183,184,185]. Caenophidia is the most species-rich and diverse group, including more than 3100 species [186]. Scolecophidia contains approximately 400 species of blind snakes with a worm-like body shape and fossorial lifestyle, and Henophidian snakes comprise about 200 species [186]. The largest family is Colubridae, commonly termed colubrids [187,188], and is the most extensively studied for cytogenetic investigation. We observe that colubrids exhibit higher variability in chromosome number and genome size compared with those of other snake families, such as Boidae and Viperidae. (Figure 4). This high degree of variation might have contributed to the remarkable diversity and speciation of colubrids. The diploid chromosome number across all snakes is 2*n* = 24–56 [152,189]. Variation involving macro- and microchromosome numbers have been reported in different families across snake lineages [152,189]. However, phylogenetic reconstruction reveals that the ancestral snake karyotype consisted of 2*n* = 36 chromosomes with 16 macro- and 20 microchromosomes. This is the karyotype commonly observed in the majority of snake species [189]. Female heterogamety (ZZ/ZW system) occurs in caenophidian snakes [20,23,24,25,27,28,41,44,45,46], whereas for noncaenophidian snakes (i) facultative parthenogenesis in pythons and boas leads to exclusively female progeny [190,191,192,193], (ii) inheritance of a color mutation in the ball python (*Python regius*) indicates a XX/XY sex determination [194], and (iii) a recent study suggests that a transition from ZW to XY may have occurred for *Boa imperator* and *Python bivittatus* based on male-specific genetic markers as well as transcriptomic and genomic data [41]. A report of heteromorphic ZZ/ZW sex chromosomes in the Madagascar boa based on conventional cytogenetics was recently confirmed by molecular cytogenetic methods in *Acrantophis* sp. cf. *dumerili* [47,195]; however, the sex chromosomes of many snake species remain undifferentiated, with no large morphological changes (such as Boidae and Phytonidae), and a low degree of differentiation between Z and W or X and Y sex chromosomes [41,47,196,197]. In comparison to the long-term stability of the Z chromosome across all snakes, the sex determination systems in noncaenophidian snakes are likely far less stable and more dynamic [47,196,197]. A recent study showed that the scolecophidian long-nosed worm snake (*Myriopholis macrorhyncha*) may have heteromorphic ZZ/ZW sex chromosomes, which are likely nonhomologous to sex chromosomes of caenophidian snakes [198]. The Z sex chromosome is indicated to share the same gene content across caenophidian snakes [20,153,199], without large morphological modifications [200]. By contrast, several repeats are known to be the primary source of differentiation of W sex chromosomes in caenophidian snakes [24,25,27,28,29], with highly degenerated and heterochromatic accumulations of repeats, and variable topology and degree of accumulation among species [181,201]. Notwithstanding conventional and molecular cytogenetic approaches, the snake W sex chromosome can remain undetectable for genomic content based on recent omic technology, except for the Indian cobra for which partial information on sex chromosomes is detectable [60]. The study of the Indian cobra genome provides an overall view of evolution by focusing on comparative genomics, thereby unlocking the diversity of toxin genes that lack in-depth sex chromosome investigation.

## 6. Chromosomics of Snake W Sex Chromosomes: Bridging the Gap between Genomes and Chromosomes

Snakes have unique genomic features that make them particularly interesting to study. The sequencing of snake genomes is increasing our understanding of their molecular evolution and genetic diversity. Evolutionary studies of venomous organisms provide sources of medical information to catalog venom proteins for drug and antivenom development. A decade ago, it was a major feat to sequence the first snake genome [204]. Subsequent advances in sequencing technology have made the sequencing of many more snake genomes attainable [60]. Technologies based on genome sequencing have the potential to resolve profiles of genetic differences between sexes at the nucleotide level, making it possible to reveal sex-specific loci or sex-specific genes in species where these had not previously been identified [40,44,45,46,205,206]. Recent advances in genome sequencing technology have assisted in the assembly of heterochromatic and/or low-complexity genomic regions, such as centromeres and differentiated W sex chromosomes. Current high-throughput sequencing methodologies and bioinformatic tools have replaced conventional molecular biological investigation techniques [29,60]. We currently have limited knowledge of whether the between-species variability of the snake W genomic content and repeat content is exceptional [25,27,28,29,30], as research to date among amniotes have been restricted predominantly to a small number of studied lineages, or whether it is common during sex chromosome differentiation.

Physical anchoring of chromosome sequences is required to validate a chromosome-level assembly. Once chromosome-level assemblies have been achieved for a greater number of snake species, investigation of changes in the packaging and interactions between chromosomes will contribute to an understanding of the role genome architecture has played during snake and amniote sex chromosome evolution. Technological advances in genomic sequencing, particularly long-read (PacBio; [207,208] and ultra-long-read (Oxford NanoPore Technologies; [209]) sequencing platforms, have provided exceptional improvement in scaffold sizes of genome assemblies. A combination of short- and long-read sequencing can provide chromosome-scale descriptions of repeat landscapes of sex chromosomes using all available genome sequence data from snakes [60,210], but currently available data for snakes is taxonomically limited and elucidation of the basic molecular machinery across snake lineages is required. Despite the improvement in long-read sequencing over short reads in genome assembly, new genome sequences often fail to produce ‘chromosome-level’ assemblies, where contigs represent a complete chromosome. In this regard, advances in cytogenetics and innovations in sequencing technology are useful for providing higher-resolution genome assemblies, and will be important for implementation in snake research moving forward. Without chromosome-level assemblies, the ability critically to examine evolutionary questions, including basic questions surrounding genome evolution and function, as well as adaptation and speciation, is limited [30,49,211,212]. Cytogenetic approaches, such as FISH, will help to generate physical maps to confirm experimentally the correct orientation of scaffolded genome sequences scaled into chromosomes. Such techniques will also enable the analysis of breakpoints and gene order, positioning of centromeric and telomeric sequences, and structural variation. A novel approach was developed in birds, where a set of chicken BAC clones was bioinformatically identified and empirically validated as a set of universal avian probes to anchor sequence scaffolds rapidly to chromosomes of sequenced species [110,213]. In this manner, it would be possible to anchor the increasing number of snake or other amniote genomes being sequenced. Appropriate samples can be obtained for the preparation of chromosomes [25,27,28], and these probe sets will provide high-resolution sequence arrangements on chromosomes. Some of the technical difficulties of FISH mapping include low-throughput data, limited microscopic resolution, and probe specificity for each analysis; in addition, examination of multiple loci simultaneously is technically challenging. Development of high-throughput, next-generation chromosome conformation capture technologies, such as the Hi-C approach [214], will provide more information on interactions and chromosomal conformations in three-dimensional genomic structures. Integration with cytogenetics mapping will allow orientation of the assembled contigs into chromosomes [215]. Bionano optical-mapping technology can also be used to acquire long-range data throughout the genome, which are highly suited to filling gaps and improving fragmented genomes in ways that are not possible using classical cytogenetics [216]. Despite substantial advances in the aforementioned technologies, no single genome has been completed with end-to-end chromosome assembly. Assembling the X chromosome telomere-to-telomere will resolve many gaps and long arrays of complex repetitive regions in the human genome using high-coverage ultra-long (Oxford nanopore) reads complemented with optical mapping [217]. This achievement will revolutionize the field of chromosomics, and high-resolution data produced from combinations of these approaches will elucidate further novelties regarding evolution of sex chromosomes [218,219].

## 7. Concluding Remarks

Sex chromosomes were discovered by Nettie Stevens in 1905. She observed that in mealworms, male cells carried chromosomes that were smaller than the rest, whereas female cells carried equally sized chromosomes [220]. The availability of genomic data for many nonmodel species, and the development of methods to detect sex-linked sequences in species with both differentiated and undifferentiated sex chromosomes, have provided a global overview of the diversity of sex-determining systems in amniotes. Recent progress on the evolution of sex chromosomes in several amniotes has supported long-standing hypotheses and, for many other amniotes, has revealed that there is no single narrative for how these regions form and evolve. Sex chromosomes show convergent genomic signatures, suggesting broader trends in their formation. The hypothesized scenario of a super-sex chromosome in the ancestral state of amniotes is followed by multiple fission to form products in the evolutionary lineages. Unpredictably, the snake W sex chromosome shows the remnants of sex chromosomal linkage homology shared among amniotes, as well as large abundances of satellites and TEs. The snake W sex chromosome may retain the most ancestral state from an ancestral super-sex chromosome in amniotes. Homologous sex chromosome turnover might occur in small clades under pressure of selection. However, the diversity of sex chromosomes reveals a remarkable number of exceptions and, therefore, a parallel diversity of underlying mechanisms. The evolutionary trap is another potential hypothesis, followed by turnover of homologous sex chromosomes in species with homomorphic sex chromosomes. Nonhomologous turnover could alternatively maintain ancestral heterogamety (that is, XY to XY transitions, or ZW to ZW), or induce a shift in heterogamety (XY to ZW, or vice versa). Although the incredible diversity of sex chromosomes and sex-determining systems has been revealed, much less progress has been achieved in understanding the evolutionary forces that have shaped this diversity. Studying ongoing or extremely recent turnovers and the possibility of a super-sex chromosome is therefore required to elucidate the causal turnover mechanisms further. A deeper characterization of sex determination in clades, where both homomorphic and heteromorphic sex chromosomes are present, will help to determine the differentiation and conservation of sex chromosomes. Studies of snake sex chromosomes of recent origin may also provide data on the formative processes, although such studies are extremely difficult given that divergence between sex chromosomes is slight. Snakes are an excellent model with which to examine hypotheses of sex chromosome evolution, which can occur rapidly; thus, population-based approaches are useful for understanding the mechanisms and patterns involved. Cytogenetic studies have presented the first glimpses of ancestral amniote super-sex evolution; however, integration of multiple NGS platforms is required to attain an in-depth understanding. The novel procedure of telomere-to-telomere assembly will further enable the mechanisms involved in reshaping sex chromosome evolution to be deciphered.

Box 1 **What is the super-sex chromosome hypothesis? What can we learn from snake sex chromosomes?**

The term “super-sex chromosome” was first proposed by Ezaz et al. [18] to express an ancestral donor source of sequences contributing to the evolutionary diversification of amniote sex chromosomes. The hypothesis of the existence of an ancient super-sex chromosome emerged from the results of extensive cytogenetic studies carried out in several amniote species, as a means of understanding the organization of candidate BACs and repeated element sequences mapped on sex chromosomes [25,28,30,58]. This hypothesis has also been postulated in genome-wide SNP studies to identify sex-specific regions [31,32] to suggest that the sex chromosomes of diverse amniote lineages exhibit sequence homology, and that a homologous super-sex portion might exist on an ancient super-sex chromosome that experienced several rearrangements including multiple fissions and repeat element insertions [28,30]. Snake sex chromosomes offer an excellent model, exhibiting a ZZ/ZW sex chromosome system, with different phases of evolutionary degeneration or amplification of the W chromosome [20,21,22,23,24,25,28,29,30,32]. To test this hypothesis, we mapped different BAC sequences on the snake W sex chromosome found to be partially homologous to other amniotes [25,28]. Recently, comparative cytogenetic analysis has identified homologies of sex chromosomes across ancestral (Henophidia) and more recent (Caenophidia) snakes [202]. The principal concept of a super-sex chromosome hypothesis is based on cytogenetics mapping; however, whether the evidence of partial homology may be exclusively linked to a super-sex segment remains unclear, with the possibility of random repeated element distribution throughout the genome. The mapped sequences, which form the basis of the original hypothesis, also represent a set of candidate loci that span a very small portion, as a few bp to several kb of the genome, compared to the total genome content of sex chromosomes that spans several megabases in amniotes. Therefore, considering this limitation, we propose to further explore the super-sex chromosome hypothesis using modern tools including genome-wide mapping of whole-sex chromosomes among diverse amniote lineages.

## Figures and Tables

**Table 1:** Comparative list of repeat contents localized on the Indian cobra Z and W sex chromosomes and the relative abundance of each identified element. Note: For certain TEs (PiggyBac and Mirage, *P*-element), the percentage was considered negligible and rounded to 0 (less than 0.01%).

Repeat Type.	Number of Copies on Z	Number of Copies on W	Size of Repeats on Z	Size of Repeats on W	Percentage of Repeats on Z	Percentage of Repeats on W
SINEs	12,030	1914	29,685,560	232,635	0.74	0.45%
Penelope	3544	2	1,145,026	313	0.32	0.00%
LINEs	50,308	8362	501,803	4,470,348	14.52	8.57%
CRE/SLACS	1	0	22,445,088	0	0.015	0
L2/CR1/Rex	35,523	7545	73	3,881,653	9.99	7.44%
R1/LOA/Jockey	545	7	15,451,390	2287	0.04	0.00%
R2/R4/NeSL	1168	27	67,375	14,578	0.35	0.03%
RTE/Bov-B	2715	435	544,043	216,332	0.61	0.41%
L1/CIN4	6313	318	936,854	351,501	3.07	0.67%
LTR elements	9461	784	4,741,345	1,335,868	3.94	2.56%
BEL/Pao	491	10	132,589	9918	0.09	0.02%
Ty1/Copia	688	40	352,463	44,578	0.23	0.09%
Gypsy/DIRS1	5955	691	5,156,575	1,248,483	3.34	2.39%
Retroviral	1563	40	335,396	31,056	0.22	0.06%
hobo-Activator	10,245	333	865,452	48,804	0.56	0.09%
Tc1-IS630-Pogo	4840	400	1,335,823	327,229	0.86	0.63%
PiggyBac	41	0	1673	0	0	0
Tourist/Harbinger	306	4	26,218	715	0.02	0.00%
Other (Mirage, P-elements, Transib)	93	0	4670	0	0	0
Unknown	888	27	95,802	6798	0.06	0.01
Small RNA	205	75	13,811	9259	0.01	0.02
Satellites	482	5	54,431	916	0.04	0
Simple repeats	44,168	25,179	2,068,877	1,322,998	1.34	2.54
Low complexity	7442	3280	469,011	234,151	0.3	0.45

## Supplementary Materials

The following are available online at https://www.mdpi.com/2073-4409/9/11/2386/s1, Supplementary note 1: Gene ontology enrichment analysis and comparative repeatomic landscaping of Indian cobra Z and W chromosomes [60,221,222,223], Supplementary dataset 1: Complete list of gene ontology terms together with function descriptions for significantly enriched genes on the W chromosome detected in the enrichment analysis.
